# Notch signaling sculpts the stem cell niche

**DOI:** 10.3389/fcell.2022.1027222

**Published:** 2022-12-20

**Authors:** Ana-Maria Zamfirescu, Andriy S. Yatsenko, Halyna R. Shcherbata

**Affiliations:** ^1^ Gene Expression and Signaling, Institute of Cell Biochemistry, Hannover Medical School, Hannover, Germany; ^2^ Mount Desert Island Biological Laboratory, Bar Harbor, ME, United States

**Keywords:** stem cell niche, cell differentiation, Delta, Notch, cancer, organoids, cell reprogramming

## Abstract

Adult stem cells depend on their niches for regulatory signaling that controls their maintenance, division, and their progeny differentiation. While communication between various types of stem cells and their niches is becoming clearer, the process of stem cell niche establishment is still not very well understood. Model genetic organisms provide simplified systems to address various complex questions, for example, how is a stem cell niche formed? What signaling cascades induce the stem cell niche formation? Are the mechanisms of stem cell niche formation conserved? Notch signaling is an evolutionarily conserved pathway first identified in fruit flies, crucial in fate acquisition and spatiotemporal patterning. While the core logic behind its activity is fairly simple and requires direct cell–cell interaction, it reaches an astonishing complexity and versatility by combining its different modes of action. Subtleties such as equivalency between communicating cells, their physical distance, receptor and ligand processing, and endocytosis can have an effect on the way the events unfold, and this review explores some important general mechanisms of action, later on focusing on its involvement in stem cell niche formation. First, looking at invertebrates, we will examine how Notch signaling induces the formation of germline stem cell niche in male and female *Drosophila*. In the developing testis, a group of somatic gonadal precursor cells receive Delta signals from the gut, activating Notch signaling and sealing their fate as niche cells even before larval hatching. Meanwhile, the ovarian germline stem cell niche is built later during late larval stages and requires a two-step process that involves terminal filament formation and cap cell specification. Intriguingly, double security mechanisms of Notch signaling activation coordinated by the soma or the germline control both steps to ensure the robustness of niche assembly. Second, in the vast domains of mammalian cellular signaling, there is an emerging picture of Notch being an active player in a variety of tissues in health and disease. Notch involvement has been shown in stem cell niche establishment in multiple organs, including the brain, muscle, and intestine, where the stem cell niches are essential for the maintenance of adult stem cells. But adult stem cells are not the only cells looking for a home. Cancer stem cells use Notch signaling at specific stages to gain an advantage over endogenous tissue and overpower it, at the same time acquiring migratory and invasive abilities to claim new tissues (e.g., bone) as their territory. Moreover, *in vitro* models such as organoids reveal similar Notch employment when it comes to the developing stem cell niches. Therefore, a better understanding of the processes regulating stem cell niche assembly is key for the fields of stem cell biology and regenerative medicines.

## Dialects of Notch signaling

The Notch signaling pathway was discovered over a century ago and received its name after the notched and serrated wing phenotype displayed in *Drosophila melanogaster* mutants (Metz and Bridges, 1917). Since then, extensive research revealed a slew of complexity and versatility surrounding this highly conserved pathway. It was found to be involved in a wide range of behaviors and developmental key points. Canonical Notch signaling is activated by interactions between the Notch receptor and Delta- or Serrate-like ligands that are expressed by adjacent cells. Upon binding, the Notch is cleaved, which leads to the release of the Notch intracellular domain (NICD). Once freed, the NICD enters the nucleus where it forms transcriptional complexes with other important participants (e.g., nuclear effector Mastermind) to activate target genes. The targets encode basic helix–loop–helix proteins that function as nuclear effectors of Notch signaling to regulate the transcriptional activity of multiple genes. As in many other pathways, the Notch pathway has more paralogs for each of the key proteins in mammals, compared to invertebrates such as *Drosophila*, which has only one Notch receptor and two ligands, Delta and Serrate (Maine et al., 1995; Lissemore and Starmer, 1999). Importantly, the core logic of the pathway is preserved and operates under the same principle throughout the animal kingdom. The direct signal transducing mechanism from the membrane to the nucleus without second-messenger amplification and regulation enables Notch to function as a juxtacrine signaling pathway to effectively regulate cell fate specification depending on the inputs from the neighboring cells (Yamamoto et al., 2014; Kovall et al., 2017). Various intra- and extracellular signals and mechanistic cues modify the strength of Notch pathway activity, which dynamically guides cells along opposing developmental fates in a tissue- and time-dependent manner (Bray, 2006).

**FIGURE 1:**
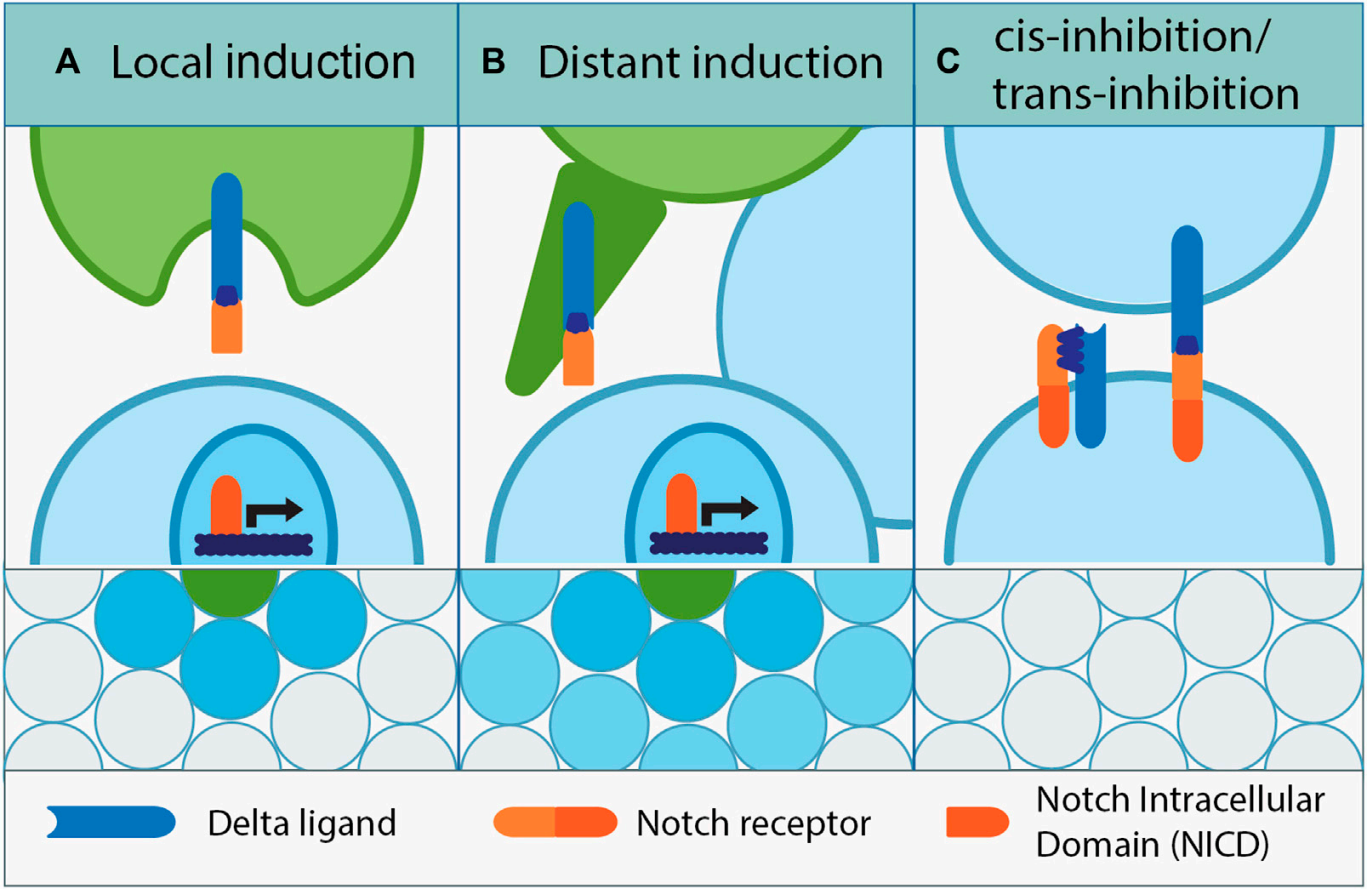
Dialects of Notch. **(A)** Among equipotent adjacent cells, a signal-sending cell induces Notch signaling in its neighbors through “lateral inhibition.” The same principle among non-equipotent cells is called “local induction.” Upon binding to the Delta ligand, the cleaved NICD domain of the Notch receptor travels to the nucleus and activates Notch-dependent gene expression. **(B)** Delta signal can also be sent further away by using cell projections, which establish direct contact with the target signal-receiving cell. This mechanism called “distant induction,” and it facilitates cell communication and Notch-dependent cell fate induction across several cell diameters. **(C)** Notch signaling inhibition can be intrinsic or extrinsic; *cis-*inhibition occurs between the Delta ligand and Notch receptor present on the same cell, while *trans-*inhibition occurs between adjacent cells that form the Delta–Notch complex, which fail to trigger Notch cleavage due to improper Delta processing or lack of sufficient membrane driven force.

Before cells acquire a certain fate, they could concomitantly express Delta or Notch and be indecisive with regard to Notch signaling. Remarkably, in a culture of equivalent cells which do not receive any outside triggers, fate determination can happen stochastically when eventually one cell would express more of either Notch or Delta due to transcriptional noise. Its neighbor would follow through by acquiring the opposite fate, and the salt and pepper pattern of Notch signaling activation would be generated at the plane level (Sprinzak et al., 2011; Shaya et al., 2017; Nandagopal et al., 2018). Notch and Delta co-expression is also the basis of a self-inhibitory mechanism called *cis*-inhibition, where Notch and Delta expressed by the same cell bind each other forming an inhibitory complex (Figure 1). This intrinsically prevents Notch signaling activation in certain cells, which opens the possibility of tissue patterning by delimitating the signal’s reach (de Celis and Bray, 1997; Sprinzak et al., 2010; del Alamo et al., 2011; Palmer et al., 2014). Inhibition can also be initiated from the surrounding cells and is coined as “*trans*-inhibition” which occurs due to the formation of trans-inhibitory Notch–Delta complexes at the membranes of adjacent cells. Insufficient mechanical force between the membranes or defective Delta endocytosis is the culprit for lack of Notch cleavage, which prevents Notch signaling activation (Kooh et al., 1993; Seugnet et al., 1997).

There are several modes of Notch signaling activation. In this case, the cells are equipotent and adjacent to each other, and they can engage in “lateral inhibition” (Figure 1) (Lai, 2004; Le Borgne, 2006; Fiuza and Arias, 2007). This simply means that in the Notch-activated cell, the ligand expression or activation is suppressed, consolidating the signal sending/receiver status of the communicating cells (Irvine and Wieschaus, 1994; Cohen et al., 1997; Rodriguez-Esteban et al., 1997; Zhang and Gridley, 1998). Lateral inhibition is meant to enforce Notch signaling boundaries and has been observed in a multitude of tissues originating from evolutionarily different species (Wilkinson et al., 1994; Kushwah et al., 2014; Petrovic et al., 2014; Vanorny and Mayo, 2017). In some cases, lateral inhibition can be also used in a long range, for example, in the epithelial cells within imaginal discs of the *Drosophila* larva. The activity of Notch signaling is mediated by Delta-promoted planar filopodia; these dynamic structures are able to intermittently transmit Delta–Notch signals for the proper spatial organization of mechanosensory bristles (Hunter et al., 2019). This mechanism not only shapes the developmental outcome, but it also has been proposed to promote tumorigenesis driven by mammalian mesenchymal cells that were prevented from differentiating through long-range lateral inhibition (De Joussineau et al., 2003; Cohen et al., 2010; Hadjivasiliou et al., 2016).

When cells are non-equipotent, Notch signaling activation is generally regarded as “peripheral induction,” which is divided once again into short and long ranges (local and distant). “Local induction” takes place when non-equipotent cells are adjacent, and the Notch signal is activated in the neighbor of a Delta-transmitting cell (Yatsenko and Shcherbata, 2018). However, when the signal needs to reach farther away, more than one cell layer apart, this is referred to as “distant induction,” accomplished through cellular projections (Figure 1), e.g., between the germline stem cells and their somatic niches (Yatsenko and Shcherbata, 2021) or tumor and mesenchymal cells in the epithelial compartment of the tumorous wing discs in *Drosophila* (Boukhatmi et al., 2020). Notably, local induction is direct, whereas the distant one can lead to gradual propagation of the signal.

Importantly, what all of the mechanisms described previously have in common is the fact that the signal activation is triggered by direct membrane contacts or through cellular projections when it is between distant cells. The intensity of the trans-interactional communication is dependent on the amount of Notch and Delta presented at the membrane level, and the activation efficiency which heavily leans on the cell–cell contact geometry (Khait et al., 2016). The reasoning behind it is that with fewer contact points there will be a lesser chance of Delta–Notch interaction, resulting in a weaker Notch activity and *vice versa*.

As important as the strength of the Notch signaling *per se* is, the early events prior to its activation also need to be carried out in a precise manner for both the receptor and ligand, as their expression, processing, and endocytosis must be spatiotemporally regulated (Le Borgne et al., 2005). For the processing of Delta, the ubiquitin E3 ligases, Neuralized (in *Drosophila* and mammals) and Mind bomb (only mammals), are needed in the signal-sending cells to promote Delta endocytosis. This step is required for Notch signaling activation because it presents the processed ligand to the surface of the signal-sending cell. Although the critical steps in Delta processing are known, it remains unclear how they could potentially be affecting the signaling strength (Perez-Mockus and Schweisguth, 2017). The signal-receiving cell has to express the Notch receptor which can bind to the ligand, forming a complex. Upon Delta–Notch ligand–receptor interaction, Notch is cleaved twice, first by a metalloprotease (Kuzbanian in *Drosophila* and ADAM10/TACE in mammals) and second by the *γ*-secretase complex, leading to the release of the Notch intracellular domain, which translocates to the nucleus, activating Notch-dependent gene expression (Pan and Rubin, 1997; Wen et al., 1997; Qi et al., 1999; Hu et al., 2002; Lopez-Schier and St Johnston, 2002).

To add to this complex tango of Delta–Notch signaling determined by the differential expression of the Notch receptor and their ligands, the cell can express factors required for Delta processing in a polarized manner. In the *Drosophila* sensory organ lineages, in one daughter cell, Notch signaling is on, while in the other one, it is off. This is a result of the polarization of the stem cell, which ensures that one daughter cell inherits the ubiquitin E3 ligase, Neuralized, and the other the Notch inhibitor, Numb. Neuralized promotes the endocytosis of Delta, thus activating Notch expression in the first daughter cell, while Numb inhibits Notch and turns up Delta expression in the second daughter cell. In this manner, cell fate is ensured and regulated through each asymmetric division (Le Borgne and Schweisguth, 2003; Fiuza and Arias, 2007). These data show that the very early fate determination of the stem cell daughters is decided through this signaling pathway by asymmetric localization of various factors and polarized endosome dynamics (Strutt et al., 2002; Langevin et al., 2005; Benhra et al., 2010; Zhao et al., 2021).

After exploring some of the main ways in which Notch signaling can orchestrate cell fate and tissue patterning, one thing is clear: the acquisition of a particular state seals the fate of subsequent neighboring cells through mutually exclusive signaling states. This translates to an elegant and malleable subsequent activation of gene-encoding factors essential in the establishment of cell identities and by extrapolation, ensuring the proper formation of tissues and organs (Lai, 2004; Fiuza and Arias, 2007; Barad et al., 2010; Hunter et al., 2016). In contrast to the emerging picture of how Notch signaling shapes cell behavior in development, there is one scenario that received little attention, that is, stem cell niche maintenance and even less, its formation. So far, it has been shown that maintenance of multiple types of adult stem cells in many organisms depends on Notch signaling (e.g., human adult muscle, brain, mammary, and hematopoietic stem cells) (Carlson et al., 2009; Ables et al., 2011; Maillard, 2014; Chakrabarti et al., 2018), suggesting that it has a conserved role in stem cell niches. More interestingly for this review, however, is that Notch signaling has been demonstrated to play an important role in formation of all well-characterized stem cell niches in *Drosophila*, such as ovarian, testicular, and intestinal niches (Ward et al., 2006; Song et al., 2007; Mathur et al., 2010; Okegbe and DiNardo, 2011). Similarly, in mammals, Notch signaling is active in the formation of various niches, e.g., the neural ventricular zone (VZ) (Byun et al., 2019). Since Notch signaling in the context of stem cell niche formation appears to have great potential but is insufficiently researched, we will focus and draw the attention to this area of interest by introducing a few fascinating examples across the animal kingdom.

## Notch and the stem cell niche formation

Around half a century ago, Schofield proposed the concept of the stem cell niche to describe the microenvironment capable of supporting stem cells (Schofield, 1978). Initially, it was used to describe independent anatomical sites observed to regulate the hematopoietic stem cell population, but later on, with the advancement of knowledge, it has been extended to multiple other tissues (Fuller and Spradling, 2007; Spradling et al., 2008). The first empirical evidence for the ability of the niche to support stemness came from one of the most studied animal models, *Drosophila*, in particular from studies of ovarian and testicular germline stem cell niches (Xie and Spradling, 2000; Tulina and Matunis, 2001). In the same vein, we will begin our exploration of Notch signaling contribution to stem cell niche establishment in the *Drosophila* ovary.

## Stem cell niches for the *Drosophila* germline stem cells

### Ovary

**FIGURE 2:**
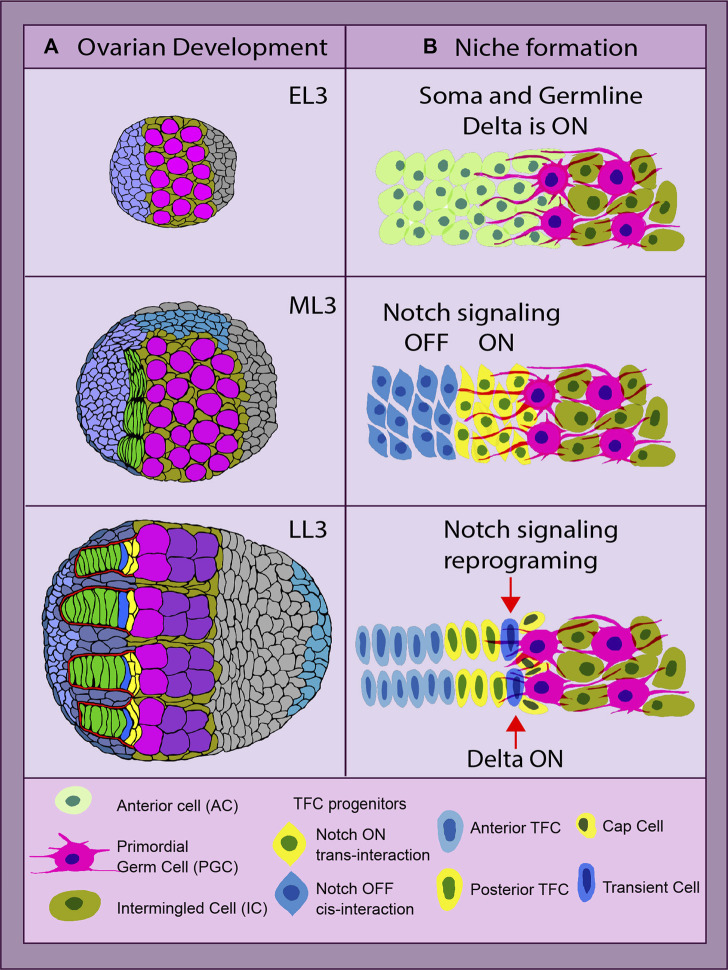
*Drosophila* ovarian development and niche formation. **(A)** depicts an overview of ovarian organogenesis throughout the early L3 (EL3) stage to late L3 (LL3), which is the last one before metamorphosis ensues. The transition at the organ level from a ball of largely undifferentiated cells to a highly complex and organized structure, with the stem cell niche consisting of the terminal filaments (TFs) (green) parallel to each other and adjacent cap cells (CpCs) (yellow). The primordial germ cells (PGCs) and germline stem cells (GSCs) are depicted in pink (Yatsenko and Shcherbata, 2018). **(B)** shows the key steps of the stem cell niche establishment. The anterior somatic cells (ACs, light green) are TF cell precursors. The anterior ACs have Notch signaling inhibited *via cis-* or *trans-*inhibition (blue), while the posterior ACs receive a Delta signal *via* the germline protrusions and become Notch activated (yellow). These two parallel mechanisms of Notch signaling status acquisition secure the first step of the stem cell niche assembly—TF formation. Later on, the most posterior of the TFC in the stalk (TC, dark blue) experiences a reprogramming of its Notch signal-receiving status, becoming a Delta-sending cell instead. With its new ability of sending the signal, it recruits the second element of the niche, the CpCs (yellow, dark nucleus), making it ready to host the stem cells. All yellow cells have Notch signaling ON, and blue cells have Notch signaling OFF (Yatsenko and Shcherbata, 2021).

Notch signaling is one of the main pathways that control the formation of the ovarian germline stem cell (GSC) niche in *Drosophila*. The formation and size of the ovarian GSC niche depend on the strength of Notch signaling since its absence leads to a reduced niche size, while Notch signaling upregulation leads to the opposite (Ward et al., 2006; Song et al., 2007; Yatsenko and Shcherbata, 2018; Yatsenko and Shcherbata, 2021). In the developing *Drosophila* ovary, there are three main elements that need to be put together in the following order to create the ovarian germline stem cell niche unit: terminal filaments (TFs), cap cells (CpCs), and GSCs (Figure 2). Notch signaling uses several ways of coordinating the process of niche assembly.

There are two major cell types that form the ovarian GSC niche *per se*: TFCs and CpCs. Despite originating from different somatic cell precursors and forming different developmental time points, both rely heavily on proper Notch signaling. However, the acquisition of a certain Notch signaling status in both cell types varies significantly and depends on the cell type, position, and the source of the Delta ligand. The formation of the GSC niche is a sequential process, where TFCs are formed first, followed by CpC specification. While both elements are part of the niche, the CpCs are the ones in direct contact with the GSCs, acting as the signaling source for their specification and maintenance (Figure 2). First, during development, the newly specified GSCs are recruited only from primordial germ cells (PGCs) located in CpC proximity. Second, only the GSCs that are physically attached to the niche are maintained in the adult germarium (Figure 2).

TFC specification begins at the third instar larva stage in the ovary that consists of anterior cells (ACs) and intermingled cells (ICs) that are intermingled with PGCs. In order to differentiate into TFCs, a certain status of Notch signaling (ON or OFF) has to be acquired by their anterior somatic cell precursors. Notch ON or OFF status in newly formed TFCs depends on their position in regard to PGCs. The cells closer to the germline are Notch active, while the cells more distant from the germline are not (Figure 2). The activation of Notch signaling in the posterior TFCs is achieved *via* PGC-generated protrusions that provide the source of the Delta ligand, resulting in the long-range Notch activation in posterior TFCs (Yatsenko and Shcherbata, 2021). In contrast to the posterior, the anterior TFCs express high levels of membrane-bound Delta, which prohibits Notch signaling activation *via* the *cis-* or *trans-*inhibition mode (Yatsenko and Shcherbata, 2021). Therefore, the commitment to TF cell fate is achieved by both Notch signaling activation and suppression. Such a dual mechanism of achieving a certain Notch status in TFC precursors provides a double security mechanism, which reinforces the first step of the stem cell niche formation, the TF assembly (Figure 2). The presence of this double mechanism is supported by the data showing that even in ovaries without PGCs acting as a Delta ligand source, the TFs and the niche, despite not being fully normal, are still being formed (Yatsenko and Shcherbata, 2021). This indicates that Delta from PGCs only plays an instructive role in this process, and the Notch signaling *cis-* or *trans-*inhibition in Notch and Delta co-expressing TF precursors is used to reassure TF formation.

The next step of ovarian GSC niche formation is CpC differentiation. CpCs originate from another somatic cell type called intermingled somatic cells (ICs), which are mixed in between PGCs. In order for CpCs to differentiate from ICs, they need to activate Notch signaling. This is achieved by Delta ligand expression by the most posterior TFC called the transient cell (TC) (Figure 2). Since the TC already has Notch signaling activated, in order to become a Delta signal-sending cell, it needs to be reprogrammed *via* steroid signaling. Steroids activate *miRNA-125* that targets Tom, a Neuralized inhibitor (Yatsenko and Shcherbata, 2018). As a result, the presence of the Neuralized allows Dl to be processed by endocytosis.

Processed Delta from the most posterior TFC binds to the Notch receptor present at the membranes of the adjacent somatic CpC precursors (ICs), leading to Notch signaling activation *via* local induction. ICs are bivalent, since they express both Notch and Delta. Notch activation *via* local induction performed by the TC resolves this bivalency, committing them to CpC fate for life (Yatsenko and Shcherbata, 2018). This concludes the process of the ovarian stem cell niche establishment.

If the expected reprogramming does not occur, the natural fluctuation of Delta will stochastically permit a cell to own the signal-sending status. Since CpC precursors co-express Delta and Notch, slightly higher levels of Delta are sufficient to induce Notch signaling in the neighboring equipotent cells *via* lateral inhibition (Yatsenko and Shcherbata, 2018). This leads to the appearance of ectopic but functional niches, underlining nature’s imperative to maintain fertility despite defects or errors.

Newly formed CpCs signal to proximal PGCs, transforming them into GSCs, which finalizes the assembly of the adult ovarian GSC niche unit. Interestingly, with various ways to activate Notch signaling in the developing ovarian GSC niche, the intensity of Notch activation is different among the cells that compose it. For example, posterior TFCs and CpCs are in close proximity but the levels of Notch activation in these cells differ, where TFCs have high Notch activity while CpCs have much lower Notch activity (Yatsenko and Shcherbata, 2021). In this case, the kinetics of Notch signaling strongly depend on the geometry of the cell contacts, the expression levels of Notch and Delta, and the range at which the Delta ligand is sent. Such various levels of Notch activity are important for the correct cell fate determination since the environment in which the niche is specified contains many bivalent cells (both ACs and ICs). Due to stochastic fluctuations, they could randomly choose Delta signal-sending status, resulting in the appearance of unnecessary ectopic niches.

We still have only witnessed the tip of the iceberg, as we know very little about the dynamics of the Notch signaling in the living cell, including aspects that are less explored, such as the role of cell mechanics in this process. Therefore, observing how Notch signaling occurs in the living organism could provide us with many new answers that will help us to better understand how the ovarian niche is formed.

On one level, distant Notch signaling governs the induction in the somatic precursors of terminal filament cell (TFC) fate by the germline-produced Delta ligand. Notch activation ensures TFC cell fate acquisition, which eventually leads to the TFC cell shape change, intercalation, and stalk formation (Figure 2). Primordial germ cells (PGCs) are able to send the Delta signal several cell diameters to the adjacent anterior somatic cells by using cellular projections (Yatsenko and Shcherbata, 2021). This makes sense, as the niche has to form in the proximity of its stem cells. What enforces the importance of the germline projections is that TFC precursors lack the ubiquitin ligase Neuralized, meaning that there will be no Delta endocytosis to apply the necessary mechanical strain to trigger the signal (Yatsenko and Shcherbata, 2018). Not only that, but also these projections could be dynamic, pulling-force generators, causing through their retraction the needed mechanical cue (Hadjivasiliou et al., 2016). This explains why Notch signaling is “ON” in the posterior TFCs touched by Delta-bearing projections, while Notch is “OFF” in the anterior TFCs that have high Delta but no Neuralized. This mechanism is particularly relevant because it has been observed in various processes, from morphogenesis to tumorigenesis (Boukhatmi et al., 2020).

Taken together, these data perfectly describe the multiple Notch signaling modes, such as spatial cellular Delta–Notch *trans*- and *cis*-interactions. In time, TFCs and CpCs eventually acquire a certain Notch signaling status because of the differential Notch signaling activation. Once the ball is rolling, higher spatial organization and fate commitment are gradually gained, until the stem cell niches are formed. In the mature ovarian niche, Notch ON/OFF signaling patterns are once again needed in the life-long maintenance of the GSCs and stem cell self-renewal which are a requirement for optimal fertility. In developing and mature tissues, Notch is indispensable, a fact that will become even clearer later in this review. It makes for a very promising target for regenerative medicine, cell therapies, and cancer treatments, and there are more excellent examples to learn from.

### Testis

**FIGURE 3:**
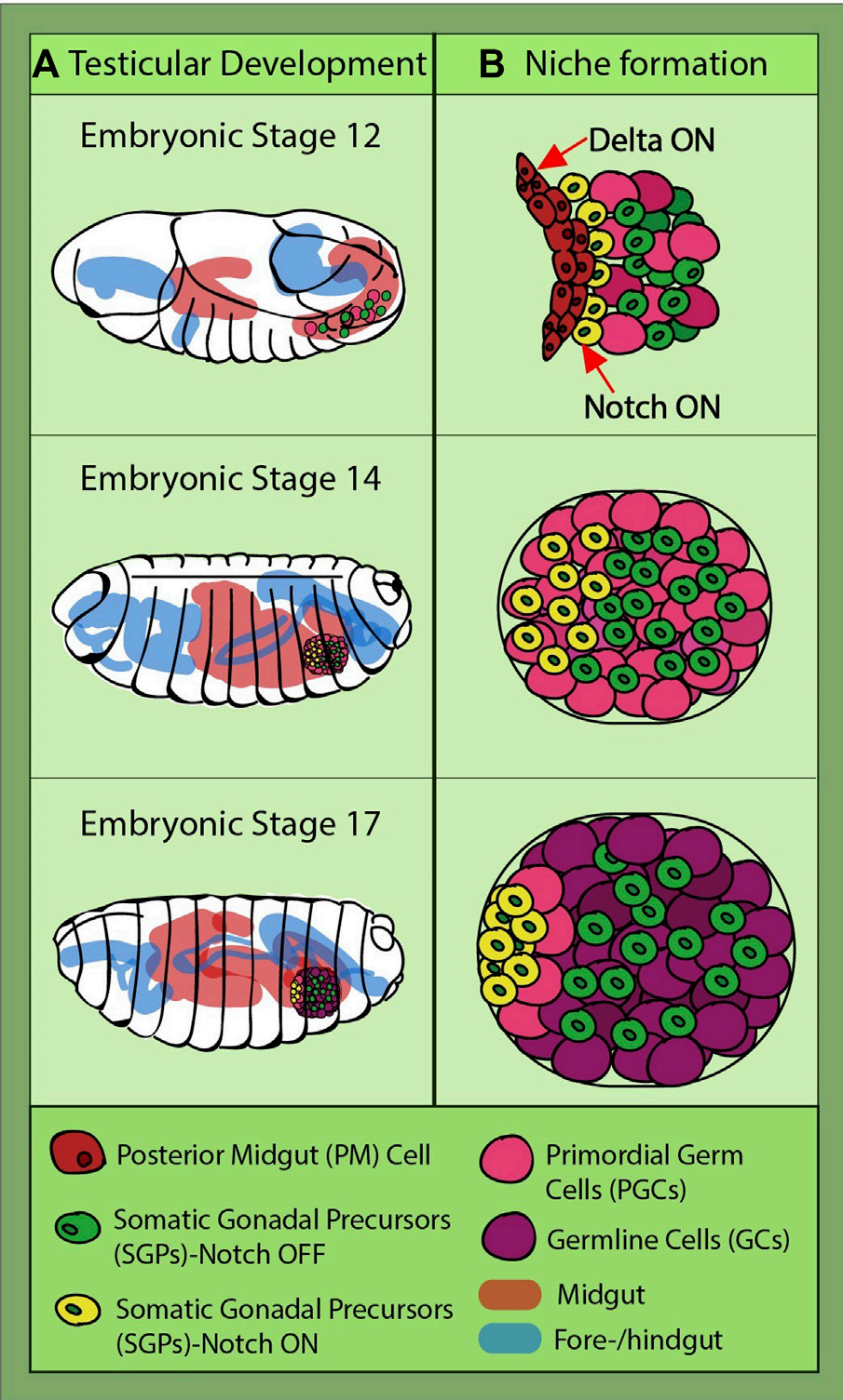
*Drosophila* testicular development and niche formation. **(A)** presents an overview of the journey of the primordial germ cells (PGCs) (pink) and somatic gonadal precursors (SGPs) (green and yellow) which takes place during embryonic stages 12–17, the last one before hatching into a larva. They are strung along together with the posterior midgut (PM); at stage 14, they leave the gut, coalesce, and go on to organize themselves into two round gonads. **(B)** depicts the direct interaction between the PM cells and some of the SGPs (yellow), this being the moment when their fate is sealed through Notch induction caused by the Delta-sending gut cells. Notch-active SGPs will go on to form the germline niche and recruit GSCs (light pink).

The ovarian stem cell niche is not the only model that can give us a glimpse into Notch involvement in niche assembly. Research in the *Drosophila* testicular niche has unveiled even earlier Notch signaling implication in the gonadal niche development. This is because the somatic gonadal precursors (SGPs) terminally differentiate at the late embryo stage, long before larval transition (Wawersik et al., 2005; Casper and Van Doren, 2006). They originate from the mesoderm, while the PGCs develop at the posterior pole of the embryo. Being initially on the outside of the embryo, the PGCs migrate through the endoderm to reach the mesodermal part called the posterior midgut (PM). While they are traveling, the SGPs are specified from the lateral mesoderm and meet with the PGCs at stage 11 at the PM (Sonnenblick, 1941; Brookman et al., 1992; Boyle and DiNardo, 1995; Boyle et al., 1997). They finally coalesce at stage 14, and at the end of the last embryonic stage, they are organized in two round gonads with an already specified niche, called the “hub” (Figure 3) (Boyle and DiNardo, 1995; Boyle et al., 1997; Jenkins et al., 2003; Clark et al., 2007). The hub consists of cells derived from a subgroup of the apical SGPs. Notch signaling in posterior SGPs is antagonized by the PGC-induced EGFR (epidermal growth factor receptor), a mechanism which restricts and ensures that only anterior Notch-positive SGPs become hub cells (Kitadate and Kobayashi, 2010).

Further studies confirmed Notch activation in a subgroup of SGPs (Okegbe and DiNardo, 2011). During their embryogenic journey from the endoderm to mesoderm, SGPs receive a Notch-inducing signal from the Delta-expressing posterior gut cells (Figure 3). A drastic reduction in hub cell numbers was observed in the *fog* mutant embryos, in which the posterior midgut is not internalized, preventing SGP communication with the Delta-expressing gut cells (Okegbe and DiNardo, 2011). Later on, the same group identified a large Maf transcription factor, Traffic jam (Tj), as the downstream target of Notch induction in hub precursors (Wingert and DiNardo, 2015). After SGPs and PGCs coalesce, in the Notch-activated SGPs, Tj is downregulated. The relatively long time (∼6 h) between Notch activation and reduction in detectable Tj levels suggests intermediary steps in this repression cascade. Reduced Tj levels lead to a relief in unpaired and fasciclin III inhibition, allowing the cells to acquire the hub cell fate. Conversely, *tj* mutants yielded ectopic niches containing cells, of which some were not completely converted to hub cells. This fits with other findings which revealed that a gene called *midline* is needed for Tj accumulation in early-stage SGPs (Tripathy et al., 2014). Interestingly, midline has been found in other tissues to antagonize Notch signaling and is being inhibited in Notch-positive cells (Das et al., 2013). This would be an interesting avenue for further exploration to determine whether midline is regulated by Notch in SGPs.

Wingert and DiNardo (2015) concluded in their experiments that Notch activates another transcription factor, Bowl, along with Tj inhibition. Bowl activation was enough to rescue the ectopic niches in *tj* mutants in terms of cell morphology, number, aggregation, and hub localization. The exact relationship between Notch and Bowl has not yet been elucidated in male gonadal development, and speculating is difficult given that their interaction is context dependent (de Celis and Bray, 2003; Hao et al., 2003; Benitez et al., 2009; Greenberg and Hatini, 2009). Nevertheless, similar to the Notch–Tj relationship, understanding Notch–Bowl interaction in greater detail would be instrumental in figuring out the subtle control mechanisms behind gonadal niche formation.

A valuable lesson that can be taken from both male and female *Drosophila* gonadal niche formation is the important role of Tj in coordinating the events, especially considering its emerging connection with Notch. This has a great translational potential, as both mammalian Tj orthologues, c-Maf and MafB, are expressed in the somatic cells dispersed between the germline in the developing mammalian gonad (DeFalco et al., 2011), while at the same time, Notch prevents differentiation of the somatic cell progenitors (Tang et al., 2008). Overall, despite our current rudimentary understanding, data highlight Notch as one of the main coordinators of gonadal stem cell niche establishment.

To enforce that idea, a recent study identified multiple signaling pathways that are active in the developing human gonad, among which was also Notch. Although the exact make-up of mammalian spermatogonial niches has not been elucidated, their cells follow a similar trajectory during development and have contact with other germ layers (Zamboni and Upadhyay, 1982; McLaren, 1991; Satoh, 1991). Single-cell RNA-seq conducted by Li et al. (2017) provided a framework to understand Notch signaling-mediated communication between human gonadal somatic cells and their stem cells, the fetal germ cells (FGCs) (Li et al., 2017). They found that in the fetal ovary and testis, two Notch ligands are highly expressed: Delta-like ligand 3 (Dll3; in FGC in all their phases of development) and Jagged1 (specifically in oogenesis). Notch 2 receptor and its target gene, HES1, were expressed in nearly all somatic cells, making their potential interaction with FGCs extremely likely. One subgroup of the somatic cells also expresses Dll3, opening the possibility of somatic inter-communication. Concomitantly, the BMP signaling pathway seems to run in parallel with Notch (Li et al., 2017). Gradually, there is a pattern emerging, especially when compared to the aforementioned gonadal stem cell niches. Although at this point, it is only speculative, it might not be a stretch of the imagination to identify similar mechanisms in the human gonadal niche in the future.

### Mammalian stem cell niches and Notch

The role of Notch signaling in the adult stem cell niches has been demonstrated in multiple systems, including mammalian models. In comparison to the straightforward Delta–Notch pathway organization in flies and worms, mammals have five Notch ligands (Delta-like 1, 3, and 4; and Jagged 1 and 2) and four Notch receptors (Bray, 2006).

For example, it has been proposed that quiescent neural stem cells (NSCs) produce their own niche cells using the Notch ligand Delta-like ligand 1 (Dll1) (Kawaguchi et al., 2013). The Dll1 protein is induced in activated NSCs and segregates to one daughter cell during mitosis. Dll1-expressing cells reside in close proximity to quiescent NSCs, which allows a feedback signal to maintain quiescent neural stem cells in the adult mouse subventricular zone, while keeping a balance between NSCs and their progeny. There are data suggesting an additional feedback mechanism *via* EGFR signaling in the progeny cells that cause cell non-autonomous Notch signaling reduction in NSCs and induction of neurogenesis in the progeny (Aguirre et al., 2010). As a result, the number of NSCs decreases, resulting in shrinkage of the stem cell pool having homeostatic and compositional altering effects on the overall tissue.

Another great example is the adult muscle satellite stem cells in which cell-autonomous Notch activity is able to induce the production of ECM collagens. These collagens are essential components of the muscle stem cell niche and are essential for the quiescence maintenance of the satellite cells. When activated, NCID targets its effector, recombining binding protein suppressor of hairless (RBP-J), and consequently upregulates enhancers close to important collagen genes (C*ol5a1*, *Col5a3*, *Col6a1*, and *Col6a2*) (Baghdadi et al., 2018a). Activation of Notch signaling in adult muscle satellite cells is required for production of the ECM collagen V (COLV), a critical component of the quiescent stem cell niche (Baghdadi et al., 2018a). Specifically, deletion of *Col5a1* leads to abnormal cell cycle entry and gradual decline in stem cells. The model proposed here has Notch as sensor of homeostatic changes and physical damage of the niche, by experiencing a sharp downregulation and pushing the stem cells out of their quiescence (Mourikis et al., 2012; Mourikis and Tajbakhsh, 2014). OFF Notch signal means less collagen V and more activated satellite cells are ready to regenerate the tissue. Interestingly, the myogenic cells only interacted with COLV when it was added to the cell medium, but not when it was a part of the coating substrate, suggesting that it acts as a signaling molecule. Searching for collagen receptors, the only cells that showed slowed down proliferation in the presence of COLV were the calcitonin receptor (CALCR)-positive cells. Subsequent experiments put CALCR downstream of the Notch-induced quiescence axis (Baghdadi et al., 2018a). When the CALCR ligand, elcatonin, was administered in Col5a1-null mice stem cells, they displayed a higher stem cell marker expression and experienced a prolongation of the G0-to-S transition (Baghdadi et al., 2018a). This means that not only satellite muscle cell state can be directly influenced, but also that CALCR adds a deeper layer of quantitative and qualitative quiescence control. Recently, it has been found that Notch is even more versatile in its activity because it ensures that the satellite cells maintain their physical position by driving the expression of *miR-708*, which targets the *Tns3* transcripts. They code for the focal adhesion component tensin-3, and its downregulation directly affects the migratory mechanisms of the satellite cells, keeping them in place (Baghdadi et al., 2018b). Of course, there are more levels of complexity regarding Delta–Notch signaling within the mammalian stem cell niches than already presented, especially when it comes to the balance modulation between stem cell self-renewal and regeneration upon injury, for example, extensively reviewed in Kann et al. (2021). However, in the scope of the present review, we focused on the role of Notch signaling in the process of stem cell niche establishment.

The more secluded intestinal crypt is a stem cell niche that, in time, attracted considerable attention, especially due to the arduous efforts of recreating it *in vitro* for different purposes. The very dynamic single-layered intestinal epithelium has a unique wave-like architecture, with invaginations called crypts and protrusions named villi. In the crypt resides a stem cell population made of highly proliferative Lgr5+ intestinal stem cells, active crypt base columnar (CBC) stem cells, and facultative stem cells. The stem cells ensure the replenishment of epithelial cells going through a rapid turnover of only a few days (Barker et al., 2007). Similar to other stem cells, they self-renew while generating transit amplifying cells that later on migrate up the villus and terminally differentiate into absorptive enterocytes, mucus-secreting goblet cells, and hormone-secreting enteroendocrine cells. The only stationary cells remaining next to the stem cells are the Paneth cells with a half-life of several weeks. In case of stem cell loss, the facultative quiescent stem cell population kickstarts their cell cycle, occupies the empty niche, and generates progeny (Bankaitis et al., 2018). Wnt/R-spondin and Notch signaling represent the primary pathways involved in intestinal cell renewal. Similar to the satellite muscle niche, Notch inhibition results in stem cell loss, niche collapse, and an amplification in the secretory cell type number (van Es et al., 2005; Riccio et al., 2008; Pellegrinet et al., 2011; VanDussen et al., 2012). However, when it comes to the crypt formation *in vivo*, Notch involvement is presently unclear. Luckily, *in vitro* models such as organoids have recently shed light on the process of intestinal niche establishment and uncovered a central role of Notch.

### Organoids

The constantly popular intestinal organoid has been a valuable tool to understand the initial events of gut development and 3D self-organization. They are created by seeding a single Lgr5+ stem cell in Matrigel which is capable of giving rise to all required cell types (Sato et al., 2009; Spence et al., 2011). However, the principles behind this self-organization are not completely understood. At first, the stem cells organize in a sphere-shaped conformation until the first so-called symmetry breaking event occurs when the Paneth cells emerge and start secreting the Wnt3a ligand (Sato et al., 2011). As the name suggests, this event is meant to polarize the structure and trigger the niche establishment.

**FIGURE 4:**
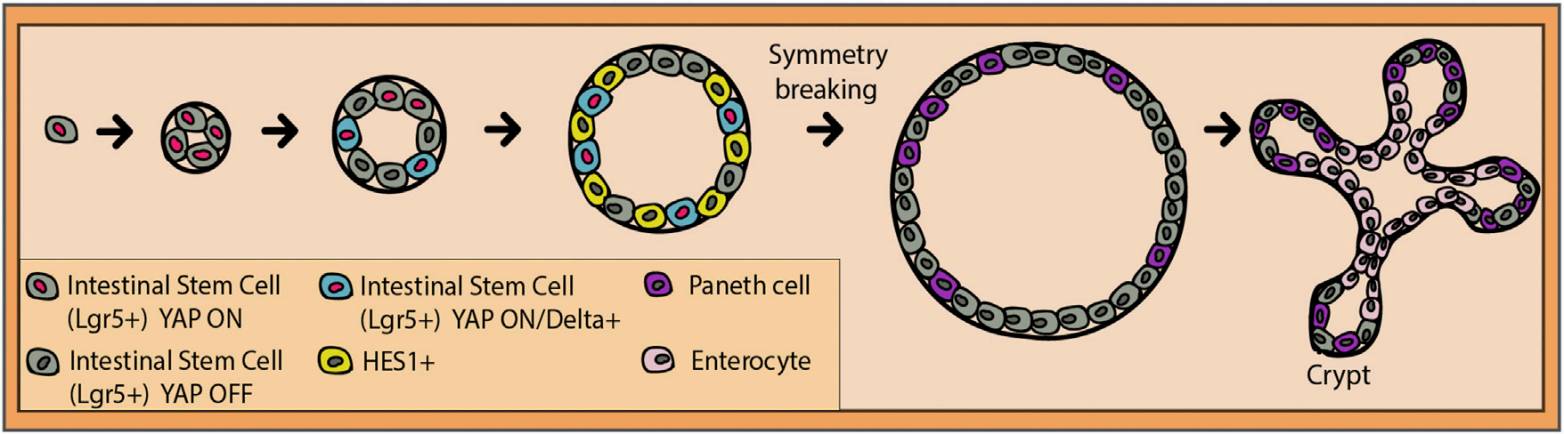
Transition from a single intestinal stem cell (gray) to an intestinal organoid. Transcription factor YAP is active in all stem cells until the four-cell stage (red nucleus). Presumably, due to physically increasing crowding of the stem cells, some of them will export YAP into the cytoplasm where it gets deactivated (dark gray nucleus). The ones that still maintain YAP ON will gradually start to express Delta (blue cells with red nucleus), and their neighbors will express a Notch target, HES1 (yellow cells with dark gray nucleus). Entering the 16–32-cell stage, the Delta-positive cells lose their nuclear YAP and are ready to commit to the Paneth cell fate. This event breaks the symmetry of the organoid and pushes the formation of the intestinal crypt (niche), where stem cells and Paneth cells reside.

When it comes to the intestinal crypt and Notch, *in vitro* studies have taught us important lessons. Serra et al. (2019) used single-cell genomics and imaging to determine the mechanisms of crypt formation. Notch activity in the organoid has been found to have two sides: symmetry breakage and cell fate maintenance (Serra et al., 2019). The latter has been previously discussed, while the former needs further attention. It seems that until the 4-cell stage, the stem cells equally present the Hippo signaling transcription factor and mechanosensor Yap1 in the nucleus (Figure 4). During the transition to the 8-cell stage, presumably due to the increased crowding of the cells, a subset of cells translocates Yap1 to their cytosol where it is inactivated (Serra et al., 2019). It was found that this Yap1 activation pattern is the one responsible for triggering symmetry breaking. This means that the cells with remaining high levels of nuclear Yap1 start to also express Dll1, which is consistent with Dll1 being a Yap1 target in other tissues (Gregorieff et al., 2015; Totaro et al., 2017). As a result, neighboring cells start expressing the Notch target, Hes1 (Serra et al., 2019). Dll1-positive cells gradually lose the nuclear Yap1 between the 16-cell and the 32-cell stages and commit to the Paneth cell fate (Figure 4). This is backed by the consequent loss of Yap1 target gene expression. To confirm these findings, *γ*-secretase inhibitors were applied, which resulted in the reduced symmetry breaking, Paneth cell differentiation, and the increase in the fraction of enterocytes (Serra et al., 2019). Overall, Notch/Dll1 activation represents the necessary nudge for the Paneth cell fate acquisition for symmetry breaking and later on, for the coordination of distinct cell fate specifications. Additionally, recent findings show the importance of the Yap ON/OFF and Notch ON/OFF circuitry in the spatial organization of the organoid (Gjorevski et al., 2022). Apparently, Paneth cells exclusively differentiate, and niches are initialized at the meeting point between a Yap1 ON cell and its Yap1 OFF neighbor. Yap1 activity was also strongly and directly correlated with the physical spreading of the cultured cells (Gjorevski et al., 2022). This links the environmental mechanosensing ability of Hippo signaling with its alternate activity patterning and in the end, with Notch–Delta lateral inhibition, selective Paneth cell differentiation, and crypt formation.

Although less studied, the organoid model of cervical cancer also showed sensitivity for fluctuating Notch levels. It is mimicking the endo-ectocervical transition zones where up to 90% of cervical cancer originates (Burghardt and Ostor, 1983; Deng et al., 2018). Therefore, this model organoid was created to enable detailed studies between inflammation induction (e.g., HPV infection), the appearance of abnormal changes in the niche and cancer occurrence. In that transition region resides a population of ectocervical stem cells which maintain complex interactions with their adjacent stromal niche cell subpopulations (Lohmussaar et al., 2021). A gene expression screen revealed high expression of the Notch ligands Dll3 and manic fringe (MFNG), while differentiated cells presented high expression levels of Notch 2 and 3 receptors, and their targets, HES1 and presenilin 1 (PSEN1) (Chumduri et al., 2021). The trans-activating interaction encourages differentiation and spatial complexity in the organoid, a fact sustained by the negative effect that *γ*-secretase inhibition has on organoid architecture (Chumduri et al., 2021). Considering all this, it is only valid to wonder if these observations also apply to cancer stem cells and their niches.

### Cancer

The concept of cancer stem cells (CSCs) has been laid out around four decades ago and describes how resident adult stem cells are pushed by various factors to assume a malignant identity and create cancerous tumors. They achieve that by hijacking the normal abilities of regeneration sustained by healthy stem cells and have been identified in many cancers (Lapidot et al., 1994; Uckun et al., 1995; Bonnet and Dick, 1997; Al-Hajj et al., 2003; Singh et al., 2004; Dalerba et al., 2007; O'Brien et al., 2007; Ricci-Vitiani et al., 2007). Despite the fact that many of these CSCs are tied to a niche, the simple act of eradicating their support as a therapeutic strategy has not yielded the expected success. Fairly recently, due to an updated and more realistic view on CSCs and their microenvironment, effective therapies have started to develop (Sun et al., 2019). Subsequent to the CSC identification, lineage tracing revealed a functional niche for some of these cells and later studies pinpointed Notch signaling as one of the active partners in the CSC dynamics across the board (Hu et al., 2012; Takebe et al., 2015).

Looking at the broader picture, early dysregulation of Notch signaling comes up as a hallmark of CSC appearance and establishment, becoming a more attractive target for all types of cancers (Farnie and Clarke, 2007; Saunders et al., 2015). Aberrant Notch induction in CSCs promotes many pro-proliferative downstream targets and helps the tumor to create a supportive environment (Androutsellis-Theotokis et al., 2006; Schreck et al., 2010; Steg et al., 2014). At times, these malignant stem cells overtake and invade the “home” of their healthy neighbors (Hu et al., 2012). We are exploring both scenarios, as Notch is a key factor that is being manipulated in cancer niche initiation and expansion.

**FIGURE 5:**
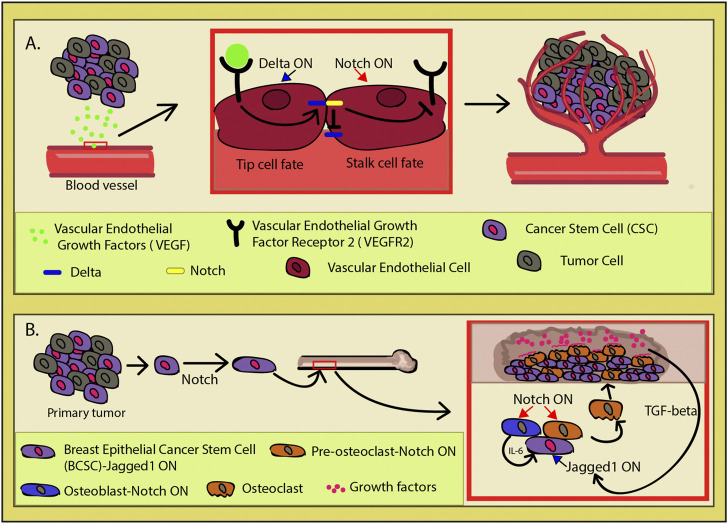
Cancer-induced angiogenesis. **(A)** Cancer stem cells (CSCs) secrete vascular endothelial growth factors (VEGFs) which induces in the nearby endothelium the process of vascular budding. VEGF activation upregulates Delta-like ligand 4 (Dll4) (blue) expression. In order to achieve functional vasculature during angiogenesis, endothelial cells need to acquire one of two cell fates (tip or stalk). These two phenotypes are necessary for a functional vasculature and exhibit different behaviors. The tip cell has migratory qualities and leads the nascent vasculature toward the VEGF source, while the stalk cells have a supportive role in the angiogenesis. Dll4 induction in an endothelial cell induces lateral inhibition in its neighbor through Notch (yellow) activation which in turn inhibits Dll4 and vascular endothelial growth factor receptor 2 (VEGFR2) expression (red square). This whole process is a two-way street, as the CSCs directly induce angiogenesis, while the newly formed vasculature network actively promotes the stem cell features of the CSCs, making it the perfect cancer niche. **(B)** In breast cancer, after the primary tumor is established, some breast epithelial cancer stem cells (BCSCs) (purple) go through biochemical changes and undergo the Notch-mediated loss of some epithelial characteristics. This transformation confers cell capacity to migrate and invade new tissues. In particular, cells have an affinity toward bone as they initiate a self-sustained program of invasion. By expressing Jagged1 (JAG1), they are able to induce Notch in the osteoblasts (blue) and osteoclasts (orange). Osteoblasts will then secrete IL-6, promoting tumor growth, while osteoclasts go into osteoclastogenesis and erode the bone, making space for the expanding mass. Bone destruction releases growth factors (pink), initiating the TGF-β pathway which upregulates JAG1 in the invading cancers cells and reinforcing the vicious cycle.

Supported in the hypoxic environment in which tumors like to sprout, the CSCs secrete the vascular endothelial growth factor (VEGF) inducing angiogenesis (Lau et al., 2017) (Figure 5A). The vascular endothelial naturally responds to this signal, and nearby blood vessels start to bud new branches and consequently create a niche to support the tumor and its CSCs. The VEGF-mediated upregulation of Dll4 or JAG1 expression in the epithelial tip cells of the budding vessel induces Notch signal trans-activation in the adjacent epithelial cell (Mailhos et al., 2001; Patel et al., 2005; Patel et al., 2006; Cao et al., 2014). This lateral inhibition represses the tip cell fate in the neighboring cell by inhibiting Dll4 and VEGF2 expressions and allowing it to gain the alternative fate, that of the stalk cell. Both types are required for proper vessel formation, as Notch inactivation promotes tip cell overproliferation, spatial disorganization, and instability of the newly formed vasculature (Mack and Iruela-Arispe, 2018). Increasing Dll4 and Notch activity leads to a sparse vasculature network, while turning it off *via* blockers had the opposite effect with the added paradoxical outcome of partially non-functional blood vessels (Noguera-Troise et al., 2006). Interestingly, it has been shown that endothelial cells directly promote the stem cell features in cancer cells through Notch signaling (Zhu et al., 2011; Lu et al., 2013; Cao et al., 2014; Ghiabi et al., 2014). Under normal circumstances, the crosstalk between VEGF and Notch is meant to coordinate and ensure the proper tube morphology and appropriate organization of the developing vasculature (Blanco and Gerhardt, 2013; Mack and Iruela-Arispe, 2018). The tumor hijacks this process for its own benefit, creating a niche capable of concomitantly sustaining cancer stem cell pool and the continuous tumor growth. This phenomenon vividly illustrates one of the complex ways in which CSCs manipulate their microenvironment through Notch.

It gets even more intriguing when Notch operates as a compass for metastatic cells to find a new microenvironment to colonize. In the breast epithelium primary tumor, hypoxia induces the loss of some epithelial characteristics *via* Jagged1(JAG1)/Notch activation in the breast cancer stem cells (BCSCs) (Al-Hajj and Clarke, 2004; Martin et al., 2005; Leong et al., 2007; Sahlgren et al., 2008; Gangopadhyay et al., 2013; Shao et al., 2015) (Figure 5B). BCSCs undergo biochemical changes and lose their epithelial cell status, exhibiting migratory capacity, invasiveness, resistance to apoptosis, and greatly elevated production of ECM components (Al-Hajj et al., 2003; Kalluri and Neilson, 2003; Scioli et al., 2019). Multiple studies demonstrate that upon Notch activation in non-invasive breast cancer cells, they gain the invasive and migratory qualities *in vivo* which is correlated with metastasis and poor prognosis (Bolos et al., 2013; Kontomanolis et al., 2014; Castro et al., 2015; Lai et al., 2018; Leontovich et al., 2018). Among multiple dysregulated factors, Notch signaling activation also causes elevation of the urokinase-type plasminogen activator (uPA), β1-integrin, β-catenin, matrix metalloproteinase (MMP)-2, and MMP-9 levels, conferring the cancer cells with even greater ability to invade new tissues (Shimizu et al., 2011; Lai et al., 2018; Wagley et al., 2020).

In metastatic breast cancer cells, *NOTCH4*, *NOTCH3*, and *JAG1* were found to be upregulated compared to other cancer cell types (Lawson et al., 2015). Particularly, *JAG1* expression was tied with bone-tropic metastatic breast cancer. SMAD-dependent TGF-β signaling induces JAG1 upregulation in the cancer cells and activates Notch 1 in osteoblasts within the niche which then secretes IL-6, stimulating the tumor growth and affecting osteoclast differentiation (Figure 5B) (Sethi et al., 2011; Wagley et al., 2020). Osteoclastogenesis is upregulated by Notch, leading to bone erosion and supporting cancer invasion through TGF-β activation. That is due to the bone being a reservoir of growth factors that are released upon osteolysis. The newly released growth factors go on to close the vicious feedback loop between the cancer cells and their newly created niche, as one of the TGF-β targets is *JAG1* (Figure 5). The cycle is reinforced by JAG1 continuous activation, which in turn amplifies its effect in the tumors’ advantage, acting as an important mediator between the bone microenvironment and freshly seeded metastatic cells (Sethi et al., 2011).

In short, the TGFβ–Notch axis enables the invasive cancer cells to discover compatible niches, and once settled in, it allows the new tumor to enhance the space it takes through perturbation and destruction of the endogenous tissue. This final point only builds on the still incomplete but fascinating ways in which this Notch signaling pathway operates in health, disease, and development.

### Final thoughts

Based on the examples illustrated in this review, Notch signaling has distinguished itself as a widespread stem cell niche sculptor. It remains to be seen if Notch signaling activation is a universal requisite for stem cell niche formation, which seems to be the tendency to which the discoveries so far point. Similarly, the concept of stem cell niches and stem cells is ever evolving, and recent studies suggest a unifying neural stemness as the ground state of tumorigenicity (Cao, 2022; Zhang et al., 2022). It draws significant parallels between tumorigenicity and pluripotent differentiation potential, both of which provide evidence for common cellular and biochemical processes (e.g., cell cycle, gene expression, and metabolism), which paints a picture of fundamental and universal mechanisms that govern the development of organisms. Notch signaling fits perfectly in this universal language as illustrated in the establishment of the stem cell niches across biological systems. From niches responsible for the maintenance of species reproduction to tissue morphogenesis and tumorigenesis, it serves as a simple but efficient and versatile communication bridge between pluripotent cells and their niches. Along with other cellular signaling pathways, the Notch pathway shapes the outcome of multicellular interactions in many different scenarios, and perhaps it is time to take a more comprehensive view of disease development and onset by learning and understanding the fundamental patterns that make up the world around us.
